# Comparative analysis of lingual versus labial orthodontics on patient comfort and treatment efficiency

**DOI:** 10.6026/973206300222107

**Published:** 2026-04-30

**Authors:** Shaik Ziauddhin, Amey Jayant Rathi, Syed Mohammad Osama Ahsan, Varun Goyal, Deepankar Bhatnagar, Priyanka Pathak, Md Kafeel Ahmed, Rubeena Naaz

**Affiliations:** 1 Department of Orthodontics and Dentofacial Orthopedics, Coorg Institute of Dental Sciences, Virajpet, Karnataka, India; 2 Department of Dentistry, MGM Medical College and Hospital, Chhatrapati Sambhajinagar, Maharashtra, India; 3 Department of Orthodontics and Dentofacial Orthopedics, Institute of Dental Sciences and Hospital, Lucknow, Uttar Pradesh, India; 4 Department of Orthodontics and Dentofacial Orthopedics, MM College of Dental Sciences and Research (MMCDSR), Maharishi Markandeshwar University, Mullana, Ambala, Haryana, India; 5 Department of Orthodontics and Dentofacial Orthopedics, Sudha Rustagi College of Dental Sciences and Research, Faridabad, Haryana, India; 6 Department of Periodontology and Implantology, MNR Dental College and Hospital, Sangareddy, Telangana, India; 7 Private Practitioner, Dental Surgeon, SRK Dental Clinic, Hyderabad, Telangana, India

**Keywords:** Lingual orthodontics, labial orthodontics, esthetic orthodontic treatment

## Abstract

Lingual orthodontic appliances meet growing esthetic demands and are replacing traditional labial systems, yet they pose challenges to patient comfort and treatment efficiency that require comparative evaluation. Therefore, it is of interest to compare lingual versus labial fixed appliances in orthodontic patients, assessing pain perception, speech impairment, tongue irritation, oral hygiene, alignment rate, visit frequency and total duration during adaptation and follow-up phases. Patient comfort parameters and treatment efficiency metrics were collected at baseline and regular intervals and analyzed statistically across both groups. Lingual appliances showed greater initial discomfort but equivalent long-term alignment and duration outcomes to labial systems with proper case selection. Thus, we report the viability of lingual orthodontics as an esthetic alternative when patient motivation and individualized selection balance early adaptation challenges.

## Background:

Orthodontic treatment has increasingly become less functional-based and more esthetic, patient-comfort and treatment-efficiency-based. As the social and professional importance of appearance increases, several patients (especially adults) want orthodontic options that are less conspicuous when treated with [1]. Traditional labial fixed appliances are the most popular modality because they are clinically efficient, versatile and cost-effective, but their appearance often makes patients unwilling to undergo treatment [2]. To address this aesthetic issue, lingual orthodontics was introduced, which places brackets on the lingual side of the teeth, making them practically invisible in normal social settings. Lingual orthodontics has been refined significantly since its development in the 1970s, with advances in bracket designs, bonding styles and computer-aided customization, making it a viable competitor to labial systems [3]. Although these developments have been made, lingual appliances have their own biomechanical and clinical complications, such as limited space to work, hard wires to adjust and increased chairside time, which can affect treatment effectiveness [4]. Patient comfort is a very important factor in orthodontic adherence and overall treatment outcome. Past research has revealed that fixed orthodontic appliances usually result in pain, mucosal irritation, speech impairment and an inability to attend to oral hygiene, especially during the non-clinical phase of the therapeutic intervention [5]. Such impacts can be exaggerated in lingual orthodontics due to direct contact between brackets and the tongue, leading to speech impairment, swallowing difficulties and tongue ulceration [6].

On the other hand, labial appliances affect only the lips and buccal mucosa and are commonly associated with faster patient adjustment and simpler oral hygiene [7]. Another aspect to consider when choosing orthodontic techniques is treatment efficiency. The alignment rate, appointment length, the need for appliance adjustment and the overall treatment time also affect the clinical workflow and patient satisfaction. Other studies claim that lingual orthodontics can be more chairside time-consuming and technically demanding and some claim that treatment time can be as time-consuming as labial orthodontics when it is better customized and delivered by inexperienced clinicians [8, 9]. Therefore, the comparative effectiveness of the two methods remains open to clinical discussion. Moreover, lingual orthodontics or labial orthodontics is a more patient-centered choice. The esthetics can be important to adults and they can tolerate short-term pain to receive a more confidential treatment choice. Still, younger patients and adolescents may be satisfied with the ease and lower cost of labial appliances [10, 11]. Therefore, it is of interest to conduct a comparative analysis of lingual and labial orthodontic methods in terms of patient comfort and efficiency of the procedure and this kind of analysis may assist clinicians to counsel patients better, to maximize the selection of appliances and to enhance the functional results and patient satisfaction in orthodontic therapy.

## Materials and Methodology:

## Study design and setting:

This paper was intended to be a prospective comparative clinical trial to determine differences in patient comfort and treatment efficiency between lingual and labial orthodontic procedures. This was done in the orthodontics department of a tertiary dental teaching hospital in a span of 18 months after the institutional review board approved the study.

## Sample selection:

The study included 60 patients who required extensive fixed orthodontic treatment. The patients were chosen according to the following criteria:

## Inclusion criteria:

[1] Age between 15 and 35 years

[2] Permanent dentition available.

[3] Mild and moderate crowding or spacing necessitating fixed appliance treatment.

[4] No previous orthodontic treatment.

[5] The patient has good periodontal health and oral hygiene.

## Exclusion criteria:

[1] Severe skeletal malocclusion with the necessity of orthognathic surgery.

[2] Temporomandibular joint disorders.

[3] Features Systemic disease of bone metabolism.

[4] Oral hygiene or compliance.

## Eligible patients were randomly divided into 2 equal groups:

[1] Group I (Lingual Orthodontics): 30 participants receiving lingual fixed appliances.

[2] Group II (Labial Orthodontics): 30 patients who have been treated with conventional labial fixed appliances.

## Orthodontic procedure:

Standard orthodontic records were collected in both groups, consisting of study models, intraoral photographs, radiographs and cephalometric analysis. Lingual brackets were indirectly bonded to lingual surfaces with the help of indirect bonding methods to ensure that they are placed correctly. Labial brackets were bonded with the traditional direct bonding techniques. The two groups used comparable wire arches (initial, leveling, working and finishing) to standardize the treatment progression. Adaptations were made after 4-6 weeks.

## Measurement of patient comfort:

A structured questionnaire and visual analog scale (VAS) were used to assess patient comfort. Parameters measured were:

Intensity of pain following placement of the appliances

[1] Mucosal irritation or tongue.

[2] Speech difficulty

[3] Chewing discomfort

[4] Oral hygiene difficulty

## The parameters were recorded to be:

[1] 24 hours after bonding

[2] 1 week

[3] 1 month

[4] 3 months

The VAS scores were 0 (no discomfort) to 10 (severe discomfort).

## Evaluation of the effectiveness of treatment:

The following clinical variables were used to measure the treatment efficiency:

[1] Rate of alignment determined on study models based on the Irregularity Index developed by little.

[2] Appointments length (minutes in chairside)

[3] Number of emergency visits

[4] Months of treatment total (months)

[5] Baseline measures were taken and follow-up visits were conducted until the cessation of active treatment.

## Outcome measures:

## Primary outcomes:

[1] Patient comfort scores (VAS)

[2] Speech adaptation period

## Secondary outcomes:

[1] Alignment rate

[2] Treatment duration

[3] Visits and chairside time.

## Statistical analysis:

The data were entered into a statistical program for analysis. Mean and standard deviation were obtained on continuous variables. Intergroup comparisons were conducted using independent t-tests. The comparison of changes over time was performed using repeated-measures ANOVA. Categorical variables were tested using the chi-square test. The obtained p-value was considered significant if below 0.05.

## Results:

A total of 60 patients completed the study, with 30 patients in each group. Both groups were comparable in age, gender distribution and baseline malocclusion severity (p > 0.05), indicating adequate group matching before treatment comparison. Patients treated with lingual appliances experienced significantly higher discomfort during the early adaptation phase. Speech difficulty was the most affected parameter, being more than twice as severe (110% higher) compared with labial orthodontics. Tongue irritation was 65% higher in the lingual group. All comfort parameters showed statistically significant differences in favor of labial orthodontics (Table 1 - see PDF). Discomfort scores declined progressively in both groups. Lingual orthodontics showed a 56% reduction in discomfort by 3 months, indicating patient adaptation over time. Differences between groups became statistically insignificant after 3 months, suggesting that initial discomfort is temporary (Table 2 - see PDF). Treatment efficiency outcomes were largely comparable between the two groups. Alignment progress differed by only 5% and total treatment duration showed no statistically significant difference. However, lingual orthodontics required 31% longer chairside time and 33% more emergency visits, reflecting increased technical complexity (Table 3 - see PDF).

## Discussion:

The current comparative analysis assessed patient comfort and treatment effectiveness between lingual and labial orthodontic appliances. The results showed that both systems were effective in ensuring satisfactory alignment. Still, there were some differences in patient- reported comfort levels, speech adaptation, difficulty with oral hygiene and time on active treatment. In this research, patients using labial appliances reported experiencing much less initial discomfort (32%) than those using lingual appliances (61%). This has been observed to be consistent with prior clinical reports showing that lingual brackets commonly cause more tongue irritation, phonetic changes and mucosal trauma during the first weeks of treatment than other bracket types, due to their location on the lingual tooth surface [12]. Research indicates that speech impairment can also occur in up to 60% of patients in the initial month of lingual therapy, with adaptation usually taking 34 weeks [13]. In our results, it was also found that the oral hygiene difficulty was more prominent in the lingual group (58% than in the labial group (41%). This is consistent with clinical orthodontic hygiene studies indicating that lingual brackets make plaque removal difficult due to limited visibility of the lingual space and limited access to the brushes. The increase in plaque quantity can also predispose patients to gingivitis and decalcification if they fail to adhere to hygiene instructions [14].

In terms of treatment efficiency, the current study demonstrated that labial orthodontics was slightly faster in achieving alignment, with 68% of cases completed within 18 months, compared to 54% in the lingual group. This is in line with systematic orthodontic reviews, which have cited that a lingual system may take slightly longer to complete treatment due to technical difficulties in wire adjustment and indirect bonding techniques [15]. Nevertheless, other studies report similar results across the two modalities for customized lingual systems and digital planning [16]. Nevertheless, aesthetic satisfaction was significantly greater in the lingual group (82) than in the labial group (49), indicating that the primary benefit of lingual orthodontics is invisibility and psychosocial acceptance, especially among adult patients. The same satisfaction has been reported in recent studies on orthodontic satisfaction, where aesthetics played a major role in the preference for appliances [13]. Overall, the facts indicate the importance of personalizing appliance choices. Lingual orthodontics offers better aesthetic advantages and can initially reduce discomfort and the need for hygiene maintenance. Still, labial orthodontics offers greater adjustability, easier accessibility for the clinician and possibly a shorter treatment period.

## Conclusion:

The lingual and labial orthodontic systems are all effective in the achievement of the desired movement and occlusal correction of the teeth. Labial appliances are more comfortable in the early stages and marginally more efficient. In contrast, lingual orthodontics is more attractive and more satisfactory for patients with a strong aesthetic sense. The choice of appliances in use should be a patient-centered priority, with clinical complexity and practitioner expertise, not efficiency alone.
